# The Arsenic–Antimony Creek at Sauerbrunn/Burgenland, Austria: A Toxic Habitat for Amphibians

**DOI:** 10.3390/ijerph19106010

**Published:** 2022-05-15

**Authors:** Wolfram Adlassnig, Brigitte Schmidt, Franz Jirsa, Andreas Gradwohl, Caroline Ivesic, Marianne Koller-Peroutka

**Affiliations:** 1 Core Facility Cell Imaging and Ultrastructure Research, Faculty of Life Sciences, University of Vienna, Althanstraße 14, A-1090 Vienna, Austria; wolfram.adlassnig@univie.ac.at (W.A.); brigitte.schmidt@univie.ac.at (B.S.); caroline.ivesic@univie.ac.at (C.I.); 2 Department of Inorganic Chemistry, University of Vienna, Althanstraße 14, A-1090 Vienna, Austria; franz.jirsa@univie.ac.at (F.J.); andreas.gradwohl@univie.ac.at (A.G.); 3 Department of Zoology, University of Johannesburg, P.O. Box 524, Auckland Park, Johannesburg 2006, South Africa

**Keywords:** *Bombina variegata*, *Rana temporaria*, *Salamandra salamandra*, metalloid, mine drainage, mine waste

## Abstract

(1) Background: All Austrian amphibians are affected by the degradation of habitats. Mining contributes to habitat destruction by the formation of spoil heaps and mine drainage waters. In Stadtschlaining/Burgenland, antimony mining led to increased arsenic (As) and antimony (Sb) concentrations in soil and water. This study investigates a contaminated creek, still inhabited by amphibians. (2) Methods: Water and soil were analyzed along the creek and correlated with the occurrence of amphibians. (3) Results: As and Sb were increased, with up to 49,000 mg/kg As and 2446 mg/kg Sb in the soil. Up to 317 mg/kg As and 156 mg/kg Sb became bioavailable under gastric, and up to 298 mg/kg As and 30 mg/kg Sb under intestinal conditions, and were absorbed upon ingestion of soil. Larvae of *Salamandra salamandra* were found throughout the creek; survival rates were low. *Rana temporaria* occurs in the most contaminated sections but does not propagate here. *Bombina variegata* appears occasionally. Amphibians seem not to be able to detect and avoid metal or metalloid contamination. (4) Conclusion: Survival of larvae is dubious, but adult amphibians survive without apparent damage under severe metalloid contamination.

## 1. Introduction

Many amphibians are seriously endangered throughout the world [1]. In Austria, all amphibians are assessed from “vulnerable” to “critically endangered” [2] and are legally protected [3]. The main threat, however, is destruction and degeneration of habitats by construction works, agriculture and industrial land use. Mining for metals and metalloids is a particularly malicious issue, as huge amounts of mine waste are produced that are rich in toxic elements, especially dissolved metalloids and metals. Proper disposal of mine waste was rarely carried out in cases of historic mining. Mineral weathering leads to the constant release of toxic elements over many centuries [4]. Toxic elements may leach into the ground water, contaminate wells or are distributed as dust. In aquatic habitats, ore weathering may lead to a combination of extreme pH and high salinity in addition to toxicity.

Amphibians are believed to be particularly sensitive against pollution as they spend a short but crucial part of their life in the water, have a relatively permeable skin, frequently swallow particles of the substrate and are generally sensitive towards a changing environment. Thus, metal pollution is assumed to contribute to the worldwide decline of amphibians [5]. Data on metal sensitivity, however, are contradictory, whereby some species were found to be quite resistant against increased concentrations of some elements which are usually regarded as toxic, including even arsenic [6]. In a pilot study, [7] showed that some species of amphibians are able to successfully colonize habitats that are heavily affected by mining activities and host strongly impoverished biocoenoses.

**Figure 1:**
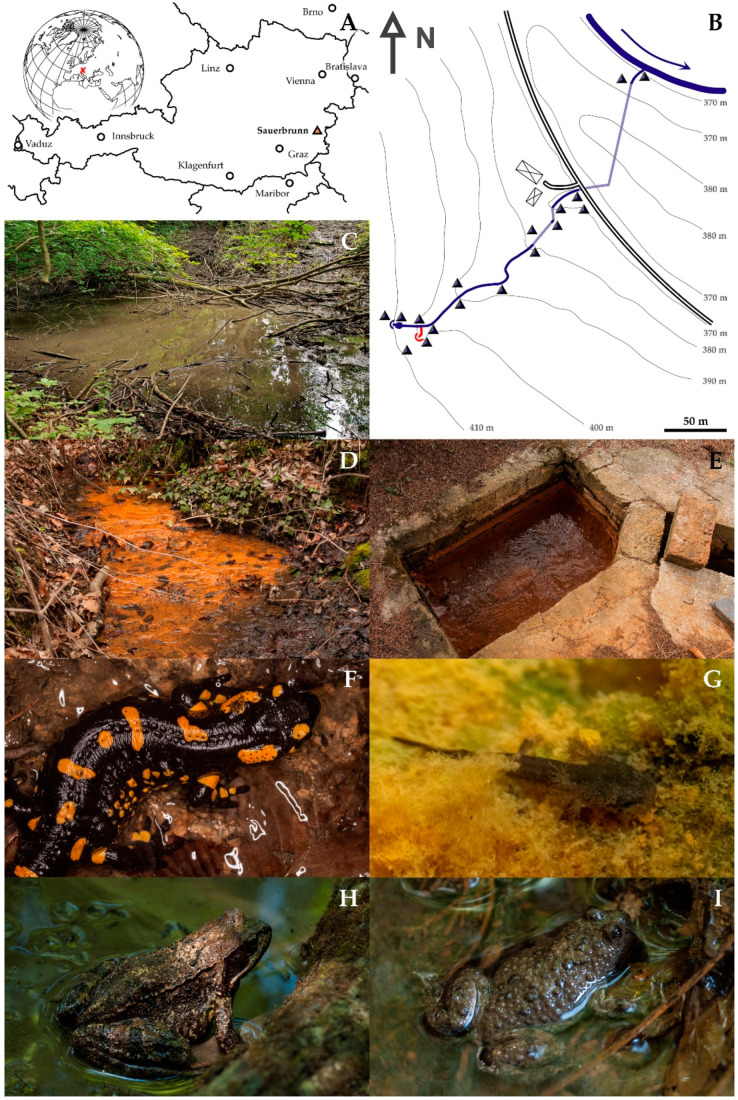
Habitat and amphibians. (**A**) Localisation of the study area within Austria/Europe. (**B**) Topographical sketch of the study area. The creek arises from a well, immediately followed by a swallow and muddy pond (**C**). 25 m below, a point source of contamination releases mine drainage water (**D**); subsequently, the creek runs through a wooded gorge and two underground passages, where the creek is accessible through two openings of the tunnel (**E**). After crossing a spoil heap, the creek reaches the floodplain of the Tauchenbach River. Dark blue: main creek and Tauchenbach River. Light blue: underground passages. Red: point source. (**F**) *Salamadra salamandra*, adult. (**G**) *Salamadra salamandra*, larva in the point source, surrounded by limonite precipitations and filamentous bacteria. (**H**) *Rana temporaria*. (**I**) *Bombina variegata*.

One of these habitats is localized in Burgenland/Austria (Figure 1A), next to the city of Stadtschlaining, where a large deposit of the metalloids antimony (Sb) and arsenic (As) was exploited for centuries [8]. After the closure of the mines in 1990, efforts were undertaken to remediate remaining spoil heaps by removal of contaminated material or by covering spoil heaps with uncontaminated soil (personal communication by local landowners). However, numerous spots can still be identified where toxic metalloids occur in concentrations increased by human activities and affect the biocoenosis [8]. The highest metalloid concentrations, both in the water and the sediment, were found in a small creek at Sauerbrunn (German for sour well), originating from a spoil heap and running into the considerably larger Tauchenbach River. However, this creek still hosts amphibians in a remarkable diversity and abundance [7].

This study investigated the Sauerbrunn rivulet (called the As-Sb Creek) and its amphibians in detail. The primary objective was to assess the diversity of amphibians. Secondary objectives included: (1) Concentrations of selected, potentially toxic elements in the water, the soil and in the sediment. (2) Availability of these elements after ingestion of soil particles. (3) Correlations of potentially toxic elements of interest with the preferred localities of the animals. (4) Evidence for increased mortality or malformation in amphibians.

## 2. Materials and Methods

### 2.1. Geoglogy and Topography of the Study Site

In the Sauerbrunn area, limestone and calcareous slates host stibnite (Sb_2_S_3_), arsenopyrite (FeAsS), pyrite (FeS2) and traces of other minerals [8,9]. These sulphidic ores, degrade to acidic and water-soluble sulphates washed out as acidic mine drainage [10]. Due to the presence of carbonates, acidic pH is rare but mobile As and Sb occur at several spots, together with increased amounts of sulphur [11]. Under aerobic conditions, As can be expected to occur as AsO_4_^3−^ [12], which is known to adsorb to goethite and other clay minerals [13,14].

**Figure 2:**
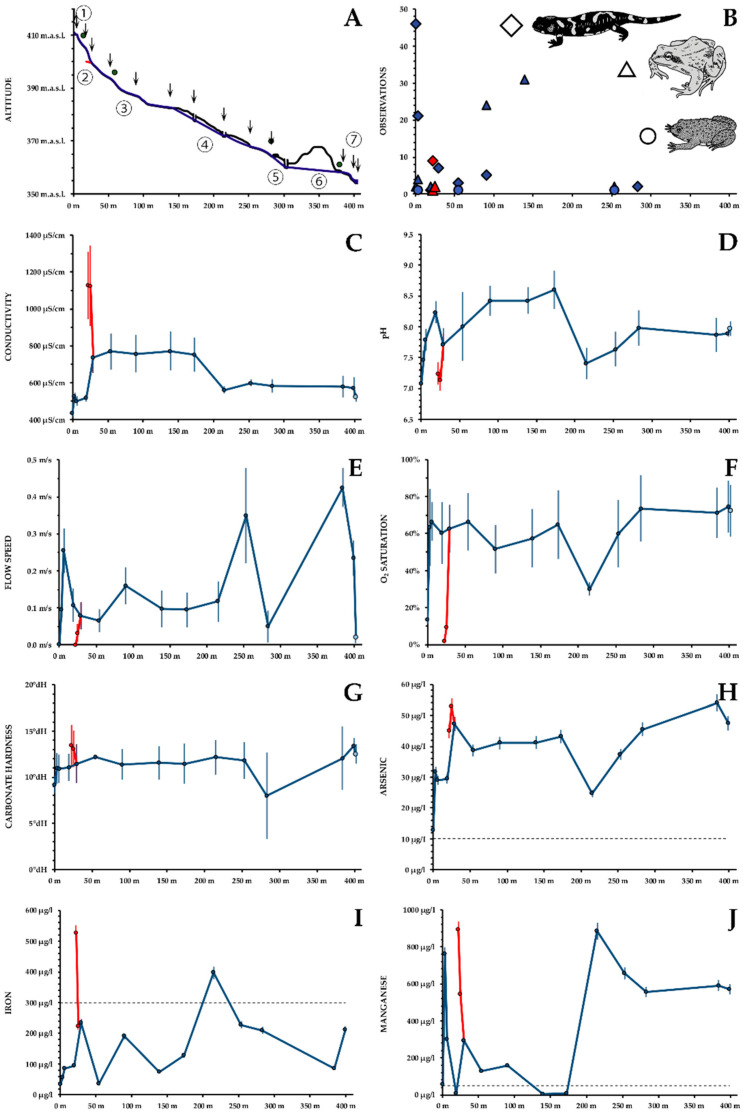
Water parameters. Blue: main creek. Red: point source. (**A**) Height profile, arrows indicate sampling (**B**) Distribution of Amphibians. ◊ *Salamandra salamandra* Δ *Rana temporaria* O *Bombina variegata* (**C**) Conductivity. (**D**) pH. (**E**) Flow speed. (**F**) O2 saturation. (**G**) Flow speed. (**H**) Dissolved As. (**I**) Dissolved Fe. (**J**) Dissolved Mn. (**A**–**G**): Mean ± standard deviation. (**H**–**J**): Measured value ± measurement error. Dotted lines indicate legal or recommended thresholds.

The As-Sb Creek runs over a course of approximately 400 m from a walled-in well through a pond and a steep valley, two underground passages and a flood plain into the Tauchenbach River (Figure 1B). The well shows little signs of contamination (Figure 1C); 25 m below the well mine drainage water is released by the point source precipitating huge amounts of limonite (Figure 1D). Figure 2A shows a height profile of the creek with the following prominent features: (1) well, immediately followed by a swallow and muddy pond with ephemeral small-scale limonite precipitation indicating influx of mine drainage water; (2) point source of contamination; (3) wooded gorge; (4) first underground passage (100 m); the creek is accessible through two openings of the tunnel (Figure 1E)—a second influx is reported by local inhabitants; (5) road; (6) second underground passage (100 m) crossing a spoil heap; (7) floodplain of the Tauchenbach River. From the well to the road, the terrain is predominantly steep and forested with *Fagus sylvatica* and some conifers (*Picea abies* and *Pseudotsuga menziesii*). The understorey is typical for a central European deciduous forest (*Hedera helix*, Araliaceae; *Oxalis acetosella*, Oxalidaceae; *Lamiastrum montanum*, Lamiaceae; etc.) without any noticeable impoverishment due to metal or metalloid contamination. Only in the surrounding of the point source, vascular plants were missing; only three species of mosses (*Rhizomnium punctatum*, Mniaceae; *Plagiochila asplenoides*, Plagiochiaceae; *Fissidens taxifolium*, Fissidentaceae) grow directly on limonite sediment. In the water of the point source, filamentous *Thiothrix nivea*-like bacteria occur (Thiothrichaceae). The flood plain is covered by a meadow with isolated ponds and puddles. The vegetation is typical for a wet meadow (*Lysimachia vulgaris*, Primulaceae; *Filipendula ulmaria*, Rosaceae; *Equisetum arvense*, Equisetaceaea; etc.) with no signs of impoverishment.

### 2.2. Sampling and Observation of Animals

Data were collected during 21 field trips between 2005 and 2021; some previously published data [7] have been included. Field trips took place throughout the active season of amphibians from March to November, in particular, from April to June. During each field trip, the As-Sb Creek was screened for amphibians, and those water parameters were analysed which were expected exhibit seasonal changes (temperature, pH, conductivity, oxygen saturation, carbonate hardness, flow speed). Supplementary Table S1 shows the number of samples analysed per sampling point.

Along the creek, 14 sampling points were selected, from the well to the influx into the Tauchenbach River. In total, 2 sampling sites were localised at a short anabranch where mine drainage water entered the creek (subsequently called the point source). At each of these sampling points, a water sample (100 mL) and a soil sample (1 kg or more) were collected in spring 2016. At 2 sampling points, limonite precipitations were present in the creek and collected as well. In this context, “soil” includes also sedimentary soils without clearly defined horizons. Four additional soil samples were taken in some distance to the creek. Topographical information was collected by using a Garmin eTrex 500 GPS device, supplemented by measuring tape and compass. Sampling points are shown in Figure 1B.

Both, amphibians in the water and at the banks of the rivulet, were recorded. During each visit, the creek was followed from the well to the Tauchenbach; the search for amphibians included turning of stones, logs, etc. within a distance of a few meters on each site of the creek. Excursions covered all seasons and all types of weather conditions; especially in spring, sampling was carried out after sunset or during rain as well.

### 2.3. Water Analysis

Except for element analysis, all measurements were performed directly in situ.

○ **Dissolved Oxygen**: titration by Aquamerck Oxygen Test 1.11107.0001.
○ **Temperature** was recorded by a conventional liquid expansion thermometer. Oxygen saturation of the water was calculated by using absolute oxygen content, altitude and temperature [15].
○ **Carbonate Hardness**: titration by Aquamerck Carbonate Hardness Test 1.08048.0001.
○ **Flow Speed**: time of the movement of a few droplets of methylene blue was measured over a defined distance.
○ **pH**: Voltcraft PH-100ATC electroden.
○ **Conductivity**: Voltcraft Pure Water Tester WA-100ATC.
○ **Metal and Metalloid Content**: samples were immediately frozen in dry ice in order to prevent precipitation of metals, and stored at <−20 °C until analysis.

### 2.4. Soil Analysis

At each sampling point, at minimum 1 kg topsoil was sampled. After drying at 80 °C, sieving and homogenisation, the <2 mm fraction was analysed. Thereby, common soil parameters and total amounts of As, Sb and selected metals were determined. Furthermore, we aimed to achieve a rough estimate of those metalloids, which would become available upon ingestion of soil by Amphibians. The digestive tract of Amphibians is composed of the same organs, and equipped with the same glands as in mammals [16]. Little information is available on the chemical composition of digestive fluids; however, gastric fluid is acidified by HCl [17], and intestinal fluid is based on bile salts [18] in frogs, indicating that digestion in Amphibians is similar to mammals. However, Amphibians are poikilothermic; thus, digestion takes place at environmental temperature. Furthermore, due to their general slower metabolism, time requirement for intestinal passage varies widely depending on temperature, size and availability of prey, but takes rarely less than 30 h [19]. Therefore, the use of an extraction protocol simulating release of toxic metals in humans appeared justified [20], whereby the sample is leached with simulated acidic gastric and alkaline bile solution at 37 °C for 1 h and 2 h, respectively. In order to account for the different physiology of Amphibians, extraction took place at room temperature over a prolonged time period (4 h for gastric and 20 h for bile solution).

○ **Soil Composition**: Humus was determined spectrometrically by reduction in K_4_Cr_2_O_7_ [21]. Sand, coarse silt, fine silt and clay were quantified by oxidation of organic compounds with H_2_O_2_, dissolution of soil aggregates by Na_4_P_2_O_7_, sieving and sedimentation [22].
○ **Soil pH**: 10 g soil were extracted overnight in 100 mL 1 M NH_4_NO_3_ under constant shaking (1 Hz). pH was measured electronically (Schott Lab 850) in the supernatant.
○ **Bioavailable Metals and Metalloids** were determined by sequential extraction, following [20]. 1 g soil was extracted with 90 mL simulated gastric juice for 4 h in order to simulate metal release in the stomach and then centrifuged (4000 rpm, 20 min). In a second step, the compounds of artificial bile solution were added in order to simulate conditions in the upper intestines; after 20 h, the sample was centrifuged again. Table 1 specifies the composition of the extractants. Digestion took place at room temperature. After each centrifugation, a sample was taken, filtered through 0.2 µm SFCA non-sterile membrane filters (Sartorius stedim Biotech, Göttingen, Germany), frozen until further analyses.

○ **Total Metalloids and Metals**: Approximately 0.5 g of oven-dried, sieved sediment samples were weighed using an analytical balance into glass tubes, and 9 mL of 34% HNO_3_ (TraceSELECT^®^ Fluka) and 1 mL of 30% H_2_O_2_ were added. Covered with air coolers, samples were processed for 2 h at 130 °C in a heating block. The leached samples were transferred to 20 mL volumetric flasks and topped up to volume using Milli-Q^®^ water. Before analyses, samples were filtered through 0.2 μm PTFE membrane syringe filters (VWR, Radnor, PA, USA). Reference samples comprising 0.2 g (dry weight) of marine sediment PACS-2 obtained from the National Research Council Canada (NRCC, Ottawa, ON, Canada) were digested and diluted in the same manner as described above.

Concentrations of other selected elements of interest (Mn, Fe, Ni, Cu, Zn, As and Pb) in sediments were determined using total x-ray reflection fluorescence spectrometry (S2 PicoFox TXRF, Bruker, Billerica, MA, USA). The instrument was equipped with a Mo source, which was run at 50 kV. Samples were measured after applying them on polished quartz slides (Bruker) and Pt was used as internal standard. Before quantification, standard-less samples were run to prove that Pt was not detectable in any of them. When lower LODs were desired, graphite furnace atomic absorption spectrometry (GF-AAS) was used, i.e., PinAAcle 900Z (Pelkin Elmer, Waltham, MA, USA). For the measurement of Sb concentration, a flame atomic absorption spectrometer AAnalyst200 (Perkin Elmer) was used. Recovery rates from the analyses of reference samples as well as limits of detection (LODs) for sediment samples are given in Table 2. Limits of detection for water analyses are given in Table 3.

### 2.5. StatisticsD

Descriptive summary statistics were calculated (arithmetic mean ± SD). Spearman rank correlation was used to test for relationships between parameters. Stata^®^ 14 (Stata Limited, College Station, CA, USA) was applied for all analyses.

## 3. Results

### 3.1. Amphibians

Three species of amphibians were observed, *Salamandra salamandra* ssp. *salamandra*, *Rana temporaria* and *Bombina variegata*. The following paragraphs show the total number of animals observed (Supplementary Table S2 shows the sightings per year).

○ *S. salamandra*: each spring, larvae were abundant in the well and the little pond below (total 67). 9 larvae were found in the well of the point source, where they were hiding between limonite precipitations and filamentous bacteria (Figure 1D,G). 14 individuals were registered in the wooded gorge, in small patches without noticeable current, 2 individuals close to entrance to the second underground passage, also in a section virtually without current. No larvae were found in the floodplain. All larvae were very small (<3 cm). No growth was observed, and their number decreased during spring. Only three fully grown terrestrial adult individuals were observed in March 2016 and May 2021, all pregnant females (Figure 1F).
○ *R. temporaria*: both subadult and adult individuals were observed (Figure 1H), but no tadpoles. The greatest abundance (59 individuals) was found in the wooded gorge. 6 individuals were at the well of the main creek and in the pond just below, 2 individuals roamed around the creek between the two underground passages. Remarkably, 4 individuals were swimming in the point source, in the most contaminated water. No *R. temporaria* was observed in the floodplain.
○ *B. variegata*: Three adult specimens of *B. variegata* were found, each time in the water (Figure 1I), one in the pond below the well, one in the wooded gorge and one between the two underground passages. Furthermore, *B. variegata* was common in the ponds of the flood plain, where also subadult individuals and tadpoles were found. These ponds exhibited slightly increased As concentrations (23.7 µg/L) and no detectable Sb.
○ Other herpetological observations in the region included *Pelophylax* spp. in fishponds next to the Tauchenbach River, but never close to the As-Sb creek. *Bufo bufo* and *Ichthyosaura alpestris* were not observed, though both species occur in the region. One subadult specimen of *Natrix natrix* was found directly in the point source in May 2016, where it was preying on frogs which were frequent at this part of the creek at that time.
○ Figure 2B shows the total number of observations for each section of the creek.

### 3.2. Water Chemistry

○ **Conductivity**: with 437 ± 22 μS/cm, conductivity is not excessively high at the main well. Until the influx of the point source, conductivity increases slightly, due to minor influx of mine drainage water. The point source shows a conductivity of 1127 ± 123 μS/cm. After mixing of the creek and the drainage water, a conductivity of 736 ± 85 μS/cm is found. This value remains quite constant until the first underground passage of the water, where an influx of uncontaminated water may occur. The resulting, lower conductivity of about 600 μS/cm remains subsequently constant, until the creek drains into the Tauchenbach with a conductivity of 522 ± 22 μS/cm (Figure 2C).
○ **pH**: Throughout the creek, neutral or slightly alkaline pH values are found. At the well, pH is 7.1 ± 0.2 and increases subsequently to 8.2 ± 0.2. The water of the point source is neutral as well (7.2 ± 0.1), resulting in 7.7 ± 0.3 after the conflux. The pH rises again to 8.6 ± 0.3. In the first underground passage pH decreases to 7.4 ± 0.3 and rises again to 7.9 ± 0.1, similar to the pH of the Tauchenbach River (Figure 2D).
○ **Flow Speed** changes throughout the creek according to the inclination. At the wells of the main creek and the point source, virtually no flow can be observed. At three small cascades, flow speed exceeds 25 cm/s (Figure 2E).
○ **Dissolved O_2_ content** shows pronounced changes. Both at the well of the main creek (concentration 2.0 ± 0.4 mg/L; saturation 14 ± 3%) and at the point source (0.3 ± 0.3 mg/L; 2 ± 2%), O_2_ is very low. Throughout the creek values of 7–9 mg/L; 60–75% are found, with the underground passage as notable exception (4.1 ± 0.5 mg/L; 30 ± 4%; Figure 2F).
○ **Hardness**: Carbonate hardness at the well is 9 ± 1°dH and increases subsequently to 12 ± 0°dH. At the point source 13 ± 2°dH are found. Within the underground passage, carbonate hardness decreases to 8 ± 5°dH, and rises subsequently to 13 ± 1°dH, similar to the Tauchenbach River (Figure 2G). Ca shows similar but more pronounced (data not shown).
○ **Dissolved As** is the main metalloid in the creek. From the well to the Tauchenbach River, the recommended concentration of 10 µg/L (WHO 2011) was consistently exceeded. The lowest concentrations was found at the main well (12.8 ± 0.6 μg/L). A few meters downstream As has risen to 31.7 ± 1.6 μg/L, obviously due to the aforementioned minor influxes of mine drainage water. At the point source, As concentrations up to 52.8 ± 2.6 μg/L was measured. After the conflux approximately 40 μg/L were found until the first underground passage. Here, As decreases to 24.7 ± 1.2 μg/L. Subsequently, As reaches 54.0 ± 2.7 μg/L, after the second underground passage, indicating a highly complex behaviour of As (Figure 2H).
○ **Dissolved Sb** is below the detection limit throughout the main creek.
○ **Dissolved Fe** is rare in the well (34 ± 2 μg/L), but occurrs in higher concentrations in the water of the pond (55 ± 3 μg/L). In the point source, an Fe concentration of 525 ± 26 μg/L is found, which decreases to 223 ± 11 μg/L over a few meters. Subsequently, Fe shows pronounced fluctuations, achieving 397 ± 20 μg/L at the first underground passage (Figure 2I).
○ **Dissolved Mn** is low at the well of the main creek (59 ± 3 μg/L), but peaks in the pond below (761 ± 38 μg/L) and in the point source (893 ± 45 μg/L). At the lower end of the wooded gorge, before the first underground passage, Mn concentrations are below the detection limit. Within the passage, Mn reaches 884 ± 20 μg/L, with values about 600 μg/L until the Tauchenbach River. Throughout most of the creek, Mn concentrations exceed the threshold of 50 μg/L (Council of the European Union 1998; Figure 2J).
○ **Dissolved Ni** is rare at the well of the main creek (6.0 ± 0.3 μg/L). At the point source 84 ± 4 μg/L are found, resulting in 35 ± 1.8 μg/L after the conflux. Ni concentrations are occasionally above the threshold of 70 μg/L (WHO 2011).
○ **Other metals** such as Cu, Pb and Zn were found in concentrations below threshold values for toxicity, or even below the detection limits.

### 3.3. Creek Sediment

At the point source and the first underground passage, the bottom of the creek is covered by orange precipitations identified as limonite (predominantly Goethite, FeO(OH) with a varying ratio of oxide to hydroxide, see [11]). Below the point source and below the first underground passage, filamentous bacteria (cf. *Thiothrix nivea*), possibly sulphur oxidisers, grow abundantly between the precipitations (Figure 1G). At the point source, an As content of 49,000 ± 9974 mg/kg was found, which decreases to 5349 ± 267 mg/kg within the first underground passage. Sb decreases from 2446 ± 122 mg/kg at the point source to 48 ± 2 mg/kg at the first underground passage.

### 3.4. Alluvial Soil

**Figure 3:**
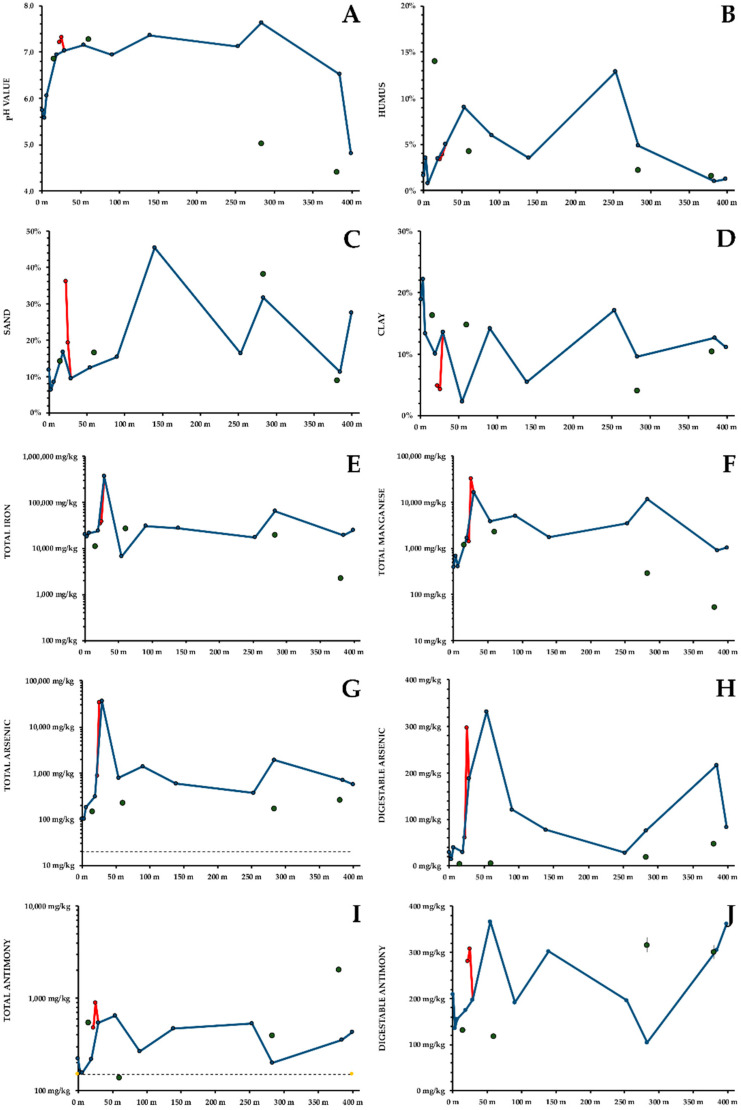
Soil parameters. Blue: banks of main creek. Red: point source. Green dots: sampling sites in some distance to the creek. (**A**) Potential pH. (**B**) Humus content. (**C**) Sand. (**D**) Clay. (**E**) total Fe. (**F**) total Mn. (**G**) total As. (**H**) Digestible As. (**I**) total Sb. (**J**) Digestible Sb. A-D: Mean ± standard deviation. E-J: Measured value measurement error.

○ **Soil pH** was mildly acidic to circumneutral (6–7) throughout the course of the creek (Figure 3A). An additional sampling point next to the second underground passage in a conifer stand, and the flood plain of the Tauchenbach showed a more acidic pH (5).

○ **Humus content** of the soil fluctuates significantly between 1% and 14% (Figure 3B). The humus content in the forested upper part of the creek, especially under coniferous trees, was higher than in the flood plain.
○ **Grain Size Distribution:** sand content (0.63–2 mm, Figure 3C) was high at the point source (36%), in the wooded gorge (45%), and in the flood plain (32%). Coarse silt (20–63 μm) oscillated between 30% and 45%, with higher concentrations in the flood plain and the upper part of the creek. Medium and fine silt (2–20 μm) were highest next to the pond, below the conflux and at the end of the second underground passage. Clay (<2 μm, Figure 3D) varied widely, no general trend was obvious, though the clay content was lower next to the point source.
○ **Soil Fe** is not necessarily a toxic contaminant. From the well to the floodplain, including the additional sampling points, Fe concentrations were usually between 20,000 and 30,000 mg/kg, with no obvious trend. At the point source, an Fe concentration of 361,576 ± 18,079 mg/kg was found, corresponding to a limonite content of at least 57.5%. High Fe concentrations (64,589 ± 3229 mg/kg) are also found at a tiny floodplain close to a second underground passage (Figure 3E). This coincides with an As peak for this sampling spot.
○ **Soil Mn** concentrations were comparatively low above the point source (400–700 mg/kg) but rose to 32,855 ± 1643 mg/kg at the point source. Subsequently, Mn concentrations remained high (1700–5000 mg/kg, >10,000 mg/kg at two sampling sites, Figure 3F).
○ **Soil As** was significantly increased; the recommended threshold of 20 mg/kg [23] was exceeded throughout the habitat (Figure 3G). Around the well and at the sampling points in some distance to the creek, As concentrations of 100–300 mg/kg were found. Below the point source and next to the conflux, As concentration was about 35,000 mg/kg. Below the conflux, As content was typically 400–800 mg/kg, though higher values were found twice. Both gastric and intestinal available As concentration was low above the point source, and at the sampling sites in some distance to the creek. Concentrations of gastric and intestinal available As peaked at the point source (317 ± 16 mg/kg and 298 ± 15 mg/kg), though the relative availabilities were extremely low (0.9% for both). A second peak was found 25 m downstream (286 ± 15 mg/kg and 333 ± 17 mg/kg, respectively). In the wooded gorge and below, gastric As availability was relatively constant (approximately 60 mg/kg). Intestinal availability went down to 28 ± 1 mg/kg but increased during the second underground passage and reached 216 ± 11 mg/kg in the floodplain (Figure 3H shows total digestible As). Maximum As digestibility is found where the clay content of the soil reaches its minimum.
○ **Soil Sb** concentrations were above the recommended threshold of 150 mg/kg [24] virtually throughout the habitat. Total Sb showed a similar distribution as As, but was less mobile (Figure 3I). Just below the point source, Sb concentration peaked with 902 ± 45 mg/kg. Subsequently, most Sb concentrations oscillated between 300 and 500 mg/kg. At three additional sampling points, Sb concentrations were comparable to the bank of the creek. Only little Sb was mobilised by simulated gastric solution. Gastric availability of Sb was 17.4–28.7% next to the point source, and <0.1% everywhere else. Intestinal Sb availability was >50% at most sampling points. At the well intestinal available Sb was rather low (210 ± 11 mg/kg), peaks were found up to 25 m below (309–367 mg/kg), followed by decreasing concentrations. In the floodplain concentrations of intestinal available Sb were higher (363 ± 18 mg/kg). Figure 3J shows total digestible Sb.
○ **Other metals** were only moderately increased. Soil Ni concentrations were just below the threshold of 75 mg/kg [25] at the well of the main creek and at the floodplain, but peaked below the point source (3253 ± 163 mg/kg) and again between the two underground passages (637 ± 32 mg/kg). Soil Pb concentration were well below the threshold of 300 mg/kg [25], except for the point source (242 ± 12 mg/kg). Soil Zn concentrations were predominantly close to the threshold of 300 mg/kg [25] but exceeded it only at the point source (1919 ± 96 mg/kg) and at the little floodplain close to the second underground passage (322 ± 16 mg/kg). Soil Cu oscillated between 10–279 mg/kg but showed no obvious correlation with the point source.

No significant regression or mixed linear models could be developed predicting As or Sb availability; however, Table 4 shows correlations between As and Sb and other soil parameters (only significant correlations shown). Thus, available As and Sb correlated significantly with total As and Sb, respectively. However, the proportion of available As and Sb was negatively correlated with total As and Sb, respectively. Furthermore, availability of both elements was lower in clay rich soils. In case of As, availability was also negatively correlated with Fe and Mn, whereas Sb availability was negatively correlated with humus.

## 4. Discussion

### 4.1. Availability of Potentially Toxic Elements

Furthermore, reducing conditions within the spoil heap and the first underground passage lead to the formation of soluble Fe(II), whereas at the surface oxidised and insoluble Fe(III) is generated. Part of this process seems to be mediated by filamentous bacteria (Figure 1G) resulting in the precipitation of limonite [FeO(OH)·n H_2_O] and an immediate drop of soluble Fe, especially at the point source itself and the first underground passage. Remarkably, high concentrations of soluble Fe are found in the wooded gorge, and at the influx to the Tauchenbach, where the water is running swiftly and rich in oxygen. The association of As and Sb with iron minerals is well known [26,27,28], resulting in a fast reduction in metalloid contamination of the water wherever limonite is precipitated. Other potentially toxic elements are released by the point source as well. However, Mn precipitates quickly under aerobic conditions, as do most other metalloids and metals.

Under aerobic conditions, As can be expected to occur as AsO_4_^3−^ [12]. Both ions are known to adsorb to goethite and other clay minerals [13,14].

As-distribution pattern throughout the creek can be explained by the precipitation and dissolution of limonite. Sb occurs also as SbO_4_^3−^ [29]. Though Sb concentrations in the water were below the detection limit, the high Sb concentrations in the limonite suggest the same mechanism. Dissolved metalloids and metals are expected to be 100% available to *S. salamandra* larvae and other water organisms. Metalloids and metals bound to limonite would become bioavailable upon ingestion.

The water level of the creek changes due to precipitations and the soil next to the creek is occasionally flooded. In addition to increased metal and metalloid content in the whole area, metal- and metalloid-rich limonite is deposited next to the creek. Throughout the area, As concentrations are above the threshold defined by applicable regulation, indicating the presence of mine waste in the substrate. However, only a tiny fraction of the metalloids can be extracted by water, 1 M KCl, 0.5 M NaHCO_3_, 0.01 M CaCl_2_ (simulation of root exudates) [11]; the plant availability is very low throughout the area [11]. However, conditions in the intestinal tract differs significantly. This study uses, for the first time, an extraction protocol designed for the estimation of bioavailability of metals for humans [20] in amphibians. In order to account for the different physiology of amphibians, extraction was conducted at room temperature and significantly prolonged. Bioavailability in simulated acidic gastric solution was low but very high under simulated alkaline intestinal conditions. Total bioavailability of As was 1–20%, occasionally up to 60%; bioavailability of As was 8–>90%.

Though it was not possible to develop a mathematical model predicting As or Sb availability, both elements showed a negative correlation between bioavailability clay content of the soil, further confirming the absorption of metalloids to clay minerals. Furthermore, As seemed to be immobilized by Fe or Mn, and Sb by humus.

### 4.2. Ecology of the Sauerbrunn Area

Superficially, the study area shows no obvious evidence for habitat degradation. The vegetation is typical for this kind of habitat and not impoverished. In difference to spoil heaps in other parts of the mining area in Schlaining [11] no typical metallophytes are found in spite of high metal and metalloid content of the soil. The lack of vascular plants at the point source is not necessarily due to metal or metalloid toxicity but might be caused by extreme shadowing at the deeply cut well. The microclimate is shadowy and humidic due to the location of most of the study area within a gorge. The frequent occurrence of *H. helix* is of special significance, since *H. helix* occupies the same niches as *S. salamandra* and the two species frequently occur together [30]. The flood plain, however, is exposed to full sunlight.

Terrestrial animals besides amphibians were not studied in detail; however, no apparent evidence for impoverishment of the fauna was found. In the creek, the situation was completely different. Below the point source virtually no aquatic invertebrates were observed (data not shown). Between the main well and the point source, *Gammarus* sp. and limpets were found.

### 4.3. Amphibians of the Sauerbrunn Area

Three species of amphibians were observed in the upper part of the As-Sb creek, one in the lower part.

In the study area *B. variegata* was found most frequently in the flood plain of the Tauchenbach River. Here, it inhabited puddles devoid of metals and metalloids but not the creek itself. These puddles resemble the optimal habitat of *B. variegata* [31,32]. Therefore, this population is inconspicuous. Only in isolated cases, *B. variegata* was found in the upper part of the creek, possibly roaming individuals, since the whole study area is within the typical range of *B. variegata* [32]. Furthermore, the pond close to the well of the As-Sb creek appears as a suitable summer habitat for non-spawning *B. variegata* [31,32]. Since *B. variegata* seems to visit the As-Sb creek only temporarily, it is unlikely that this population is seriously affected by metal or metalloid toxicity.

Adult and subadult *R. temporaria* are very frequent in the upper part of the As-Sb creek; superficially the habitat appears highly suitable [33]. Spawning may take place in larger inaccessible ponds outside the study area. *R. temporaria* was found both within the creek and on its banks. The highest abundancy was observed around the main well and in the lower part of the wooded gorge. However, several observations were made directly at and in the point source. Our data are consistent with the assumption that *R. temporaria* is moderately repelled by metal or metalloid rich water. *R. clamitans* and *Bufo americanus* have been found to incorporate significant amounts of metalloids both from the water and from prey [34]; thus, metal and metalloid absorption through the skin and by ingestion of soil particles can be expected in *R. temporaria* as well. Tadpoles of the toad *Anaxyrus boreas* are highly resistant towards As and Sb [35], whereas tadpoles of *R. pipiens* show signs of physiological stress after Sb exposure [6]. Since the As-Sb creek is colonised only by adult and subadult *R. temporaria* but not by tadpoles, it appears plausible that they are able to tolerate these elevated metalloid levels.

The wooded gorge superficially appears as a perfect habitat [30] for *S. salamandra*. Several adult individuals occur in the study area and spawn annually in the As-Sb creek. Though adult *S. salamandra* may ingest metal/metalloid rich soil particles and earth worms [36], massive uptake from the water is unlikely and the persistence of the population seems not to be threatened.

*S. salamandra* larvae are observed first and most abundantly at the main well. It can be assumed that spawning takes place here. Avoidance of metalloid-rich water by spawning females is possible. Some larvae were obviously drifted down streams; their frequency decreased with growing distance to the well. Within the creek larvae showed a clear preference for microhabitats without current. This included even the point source where larvae even accumulated. Here, they were neither rejected by low oxygen saturation, as already observed by Thiesmeier [30], nor by extreme metal or metalloid concentrations.

Successful metamorphosis, however, seems to be a very rare event. No evidence for larval growth was found, but all larvae seem to die at an early stage. Three possible reasons can be identified:

- The abundance of suitable prey animals is very low throughout the creek. Death of the larvae might be caused by starvation. Cannibalism might account for occasional successful metamorphosis sufficient to maintain the population.
- Metal and metalloid content of the water exceeds applicable thresholds throughout the creek. Reduced growth of *S. salamandra* larvae have been observed in waters contaminated by As and multiple metals [37], the same seems to apply to other Urodela [38]. Thus, fatal concentrations of metalloids might be absorbed via the permeable larval skin. However, larval mortality was also high above the point source.
- Ingestion of limonite particles seems unavoidable during suction-snapping. The particles are considerably smaller than potential prey and are easily dispersed. They contain high concentration of metalloids which become bioavailable under intestinal conditions. Limonite accumulates especially under those low-current conditions preferred by the larvae (Figure 1G). Furthermore, trace element concentrations in the tissue of *S. salamandra* larvae correlate poorly with concentrations in water [37], indicating that uptake from sediment might be more relevant than from water. Thus, it is suggested that ingestion of As-Sb-rich limonite is a major stressor for *S. salamandra* larvae in the creek, though they do not seem to avoid metalloid-rich microhabitats and may not be able to sense increased metalloid concentrations.

## 5. Conclusions

- High concentrations of As-Sb found in the creek, which precipitate together with limonite wherever Fe^2+^ is oxidised under aerobic conditions. Flooding deposits metalloids also at the banks.
- Metalloids become readily bioavailable under simulated intestinal conditions, making uptake of potentially toxic metalloids very likely.
- *B. variegata* and *R. temporaria* are frequent in the mining area but seem to avoid the most metal rich parts of the creek, at least to some degree.
- Larvae of *S. salamandra* exhibit a high mortality in the creek. Ingestion of As-Sb-rich limonite may be a major factor, besides absorption through the skin and starvation.

## Figures and Tables

**Table 1:** Extractants for the digestions of soil and sediment samples. All reagents in this table were analytical grade (Merck).

Gastric Solution		Bile Solution	
Pepsin	1.25 g/L	Pankreatin	45 mg/L
Sodium Malate	0.5 g/L	Bile Salt	155 mg/L
Sodium Citrate	0.5 g/L	NaHCO_3_	pH 7.0
Lactic Acid	420 µL/L		
Acetic Acid	500 µL/L		
HCl	pH 2.5		

**Table 2:** Recovery rates for the analyses of certified reference material PACS-2 by NRCC for respective methods and LODs under the applied conditions.

Element	Method	Reference Value (mg/kg)	Measured Mean ± SD (mg/kg)	Recovery Rate	LOD (mg/kg)
Mn	TXRF	440 ± 19	350 ± 26	79.5%	2.0
Fe	TXRF	40,900 ± 600	36,600 ± 420	89.8%	10.0
Ni	TXRF	39.5 ± 2.3	35.6 ± 1.7	90.0%	0.4
Cu	TXRF	310 ± 12	319 ± 21	102.9%	0.8
Zotn	TXRF	364 ± 23	107.2 ± 5.5	101.8%	2.0
As	TXRF	26.2 ± 1.5	26.8 ± 2.0	102.3%	2.0
As	GF-AAS	26.2 ± 1.5	27.5 ± 5.1	104.9%	0.008
Pb	TXRF	183 ± 8	182 ± 6	99.2%	2.0

**Table 3:** Method LODs for the respective method and element.

Element	Method	LOD (µg/L)
Mn	TXRF	30
Fe	TXRF	50
Ni	TXRF	25
Cu	TXRF	20
Zn	TXRF	10
As	GF-AAS	0.2
Sb	Flame-AAS	1000
Pb	TXRF	15

**Table 4:** Correlations between As/Sb and other soil parameters.

		Spearman’s ρ	*p*-Value
Total As	Total Fe	0.562	0.024
Total As	Total Mn	0.700	0.019
Available As (mg/kg)	Total As	0.833	<0.001
Available As (mg/kg)	Total Mn	0.606	0.008
Available As (mg/kg)	Clay	−0.515	0.029
Availability As (%)	Total Mn	−0.556	0.017
Availability As (%)	Total As	−0.538	0.021
Available Sb (mg/kg)	Total Sb	0.486	0.041
Available Sb (mg/kg)	Clay	−0.682	0.002
Availability Sb (%)	Total Sb	−0.829	<0.001
Availability Sb (%)	Humus	−0.538	0.021

## Data Availability

Not applicable.

## Supplementary Materials

The following supporting information can be downloaded at: https://www.mdpi.com/article/10.3390/ijerph19106010/s1, Table S1: Number of samples for each water parameter and sampling point; Table S2: Number Amphibian sightings per species and year.

## References

[1] Brito D. (2008). Amphibian conservation: Are we on the right track?. Biol. Conserv..

[2] Gollmann G., Bundesministerium für Land und Forstwirtschaft (2007). Rote Liste der in Österreich gefährdeten Lurche (Amphibia) und Kriechtiere (Reptilia). Rote Listen Gefährdeter Tiere Österreichs. Teil 2: Kriechtiere, Lurche, Fische, Nachtfalter, Weichtiere.

[3] Bundesgesetzblatt (Federal Law Announcement) (1983). Übereinkommen über die Erhaltung der Europäischen Wildlebenden Pflanzen und Tiere und ihrer Natürlichen Lebensräume. Bundesgesetzbl. Rep. Österr..

[4] Adlassnig W., Wernitznig S., Lichtscheidl I.K., Kothe E., Varma A. (2011). Historical copper spoil heaps in Salzburg/Austria. Geology, mining history, contamination and vegetation. Bio-Geo Interactions in Metal Contaminated Soils.

[5] Blaustein A.R., Romansic J.M., Kiesecker J.M., Hatch A.C. (2003). Ultraviolet radiation, toxic chemicals and amphibian population declines. Divers. Distrib..

[6] Chen T.-H., Jackson A.G., Karasov W.H. (2009). Chronic exposure to pentavalent arsenic of larval leopard frogs (*Rana pipiens*): Bioaccumulation and reduced swimming. Ecotoxicology.

[7] Adlassnig W., Sassmann S., Grawunder A., Puschenreiter M., Horvath A., Koller-Peroutka M. (2013). Amphibians in metal-contaminated habitats. Salamandra.

[8] Pollak A. (1955). Neuere Untersuchungen auf der Antimonerzlagerstätte Schlaining. Berg-Hüttenmänn. Monatsh..

[9] Cerny I. (1981). Geochemische Untersuchung von Karbonatgesteinen im Antimonbergbau Schlaining (Burgenland). Berg-Hüttenmänn. Monatsh..

[10] Nordstrom D.K. (2011). Mine waters: Acidic to circumneutral. Elements.

[11] Steinhauser G., Adlassnig W., Lendl T., Peroutka M., Weidinger M., Lichtscheidl I.K., Bichler M. (2009). Metalloid contaminated microhabitats and their biodiversity at a former antimony mining site in Schlaining, Austria. Open Env. Sci..

[12] Smedley P.L., Kinniburgh D.G. (2001). Source and behaviour of arsenic in natural waters. United Nations Synthesis Report on Arsenic in Drinking Water.

[13] Mamindy-Pajany Y., Hurel C., Marmier N., Roméo M. (2011). Arsenic (V) adsorption from aqueous solution onto goethite, hematite, magnetite and zero-valent iron: Effects of pH, concentration and reversibility. Desalination.

[14] O’Reilly S.E., Strawn D.G., Sparks D.L. (2001). Residence Time Effects on Arsenate Adsorption/Desorption Mechanisms on Goethite. Soil Sci. Soc. Am. J..

[15] Oehme F., Schuler P. (1983). Gelöst-Sauerstoff-Messung.

[16] Storch V., Welsch U. (2014). Kükenthal Zoologisches Praktikum.

[17] Durbin R.P. (1964). Anion requirements for gastric acid secretion. J. Gen. Physiol..

[18] Une M., Matsumoto N., Kihira K., Yasuhara M., Kuramoto T., Hoshita T. (1980). Bile salts of frogs: A new higher bile acid, 3 alpha, 7 alpha, 12 alpha, 26-tetrahydroxy-5 beta-cholestanoic acid from the bile *Rana plancyi*. J. Lipid Res..

[19] Feder M.E., Burggreen W.W. (1992). Environmental Physiology of the Amphibians.

[20] Intawongse M., Dean J.R. (2008). Use of the physiologically-based extraction test to assess the oral bioaccessiblity of metals in vegetable plants grown in contaminated soil. Environ. Poll..

[21] Kandeler E., Schinner F., Öhlinger R., Kandeler E., Margesin R. (1993). Humusbestimmung durch Naßoxidation. Bodenbiologische Arbeitsmethoden.

[22] Öhlinger R., Schinner F., Öhlinger R., Kandeler E., Margesin R. (1993). Bestimmung der Korngrößenverteilung. Bodenbiologische Arbeitsmethoden.

[23] Umweltbundesamt (2003). Zulässige Grenzwerte (Richtwerte) für Schadstoffe in Klärschlamm und Boden.

[24] Mergenthaler B., Richner T. (2002). Mobilität und geochemisches Verhalten von Antimon im Boden von Schiessanlagen. Master’ Thesis.

[25] Hein H., Klaus S., Meyer A., Schwedt G. (2016). Richt- und Grenzwerte. Teil A: Übersichten zu den Deutschen und Europäischen Richtlinien.

[26] Ackermann J., Vetterlein D., Kuehn T., Kaiser K., Jahn R. (2010). Minerals controlling arsenic distribution in floodplain soils. Europ. J. Soil. Sci..

[27] Gál J., Hursthouse A., Cuthbert S. (2007). Bioavailability of arsenic and antimony in soils from an abandoned mining area, Glendinning (SW Scotland). J. Environ. Sci. Health.

[28] Wilson S.C., Lockwood P.V., Ashley P.M., Tighe M. (2010). The chemistry and behaviour of antimony in the soil environment with comparisions to arsenic: A critical review. Environ. Poll..

[29] Mierzwa J., Mumbi R., Avedananda R., Rakshit S.E., Michael E., Sarkar D. (2021). Antimony (V) Adsorption at the Hematite–Water Interface: A Macroscopic and In Situ ATR-FTIR Study. Soil Syst..

[30] Thiesmeier B. (2004). Der Feuersalamander.

[31] Gollmann B., Gollmann G. (2012). Die Gelbbauchunke. Von der Suhle zur Radspur.

[32] Seidel B., Nöllert A. (1996). Streifzug durch die Verhaltens- und Populationsbiologie von Gelbbauchunken, *Bombina variegata* (L., 1758) (Anura: Bombinatoridae), in einem Habitat mit temporären Gewässern. Naturschutzreport. Verbreitung, Ökologie und Schutz der Gelbbauchunke.

[33] Nöllert A., Nöllert C. (1992). Die Amphibien Europas. Bestimmung-Gefährdung-Schutz.

[34] Moriarty M.M., Koch I., Reimer J. (2013). Arsenic species and uptake in amphibians (*Rana clamitans* and *Bufo americanus*). Environ. Sci. Processes Impacts.

[35] Dovick M.A., Arkle R.S., Kulp T.R., Pilliod D.S. (2020). Extreme arsenic and antimony uptake and tolerance in toad tadpoles duringdevelopment in highly contaminated wetlands. Environ. Sci. Technol..

[36] Langdon C.J., Piearce T.G., Meharg A.A., Semple K.T. (2003). Interactions between earthworms and arsenic in the soil environment: A review. Environ. Poll..

[37] Pavlović S., Krizmanić I., Borković-Mitić S., Stojsavljević A., Mitić B. (2020). A first record of the antioxidant defense and selected trace elements in *Salamandra salamandra larvae* on Mt. Avala and Mt. Vršački Breg (Serbia). Arch. Biol. Sci..

[38] Chang J.-S., Man G., Kyoung-Woong K. (2009). Effect of arsenic on p53 mutation and occurrence of teratogenic salamanders: Their potential as ecological indicators for arsenic contamination. Chemosphere.

