# Supplementary material for: The Arsenic–Antimony Creek at Sauerbrunn/Burgenland, Austria: A Toxic Habitat for Amphibians
Source: Int J Environ Res Public Health. 2022 May 15;19(10):6010. doi: 10.3390/ijerph19106010 (PMC9141369; doi:10.3390/ijerph19106010)
Supplement: Supplementary file 1 [file ijerph-19-06010-s001.zip › ijerph-1674693-supplementary.pdf]

International Journal of
Environmental Research and Public Health

MDPI

Supplement Table S1: Number of samples for each water parameter and sampling point

Sampling Point	pH Value	Conductivity	Temperature	Oxygen	Carbonate Hardness	Flow Speed
Point source (well)	8	9	11	8	7	4
Point source	6	6	8	5	3	2
Pond in meadow	5	7	7	4	3	n.d.
Main Well (0 m)	9	13	13	9	5	5
Creek (3 m)	7	11	11	7	4	5
Pond (6 m)	8	12	12	8	7	2
Creek (19 m	10	11	13	10	7	4
Influx of point source (29 m)	9	14	16	11	9	8
Creek (54 m)	9	9	11	10	5	2
Creek (90 m)	8	10	12	11	7	4
Creek (139 m)	11	12	14	12	8	4
Start of 1st underground (173 m)	5	8	8	6	4	4
1st underground passage (215 m)	3	7	7	5	4	4
End of 1st underground passage (253 m)	7	10	10	8	6	4
Start of 2nd underground passage (283 m)	12	14	13	13	9	4
End of 2nd underground passage (384 m)	8	12	10	10	6	2
Creek (399 m)	6	10	9	9	5	2
Influx into Tauchenbach (402 m)	7	11	9	9	5	2
Total	138	186	194	155	104	62

Supplement Table S2: Number Amphibian sightings per species and year

Species	Sighting	Year
Salamandra salamandra	1	2008
Salamandra salamandra	1	2015
Rana temporaria	1	2015
Bombina variegata	1	2015
Rana temporaria	20	2015
Rana temporaria	1	2015
Rana temporaria	30	2015
Rana temporaria	1	2015
Salamandra salamandra	1	2016
Salamandra salamandra	36	2016
Salamandra salamandra	1	2016
Salamandra salamandra	6	2016
Salamandra salamandra	16	2016
Rana temporaria	1	2016
Rana temporaria	1	2016
Rana temporaria	1	2016

Species	Sighting	Year
Natrix natrix	1	2016
Rana temporaria	1	2016
Salamandra salamandra	2	2016
Bombina variegata	2	2016
Rana temporaria	4	2016
Rana temporaria	1	2016
Salamandra salamandra	5	2017
Salamandra salamandra	1	2017
Salamandra salamandra	6	2017
Salamandra salamandra	4	2017
Rana temporaria	3	2017
Bombina variegata	1	2017
Salamandra salamandra	5	2017
Rana temporaria	1	2017
Bombina variegata	1	2017
